# Supplementary material for: Establishment and Social Impacts of the Red Imported Fire Ant, Solenopsis invicta, (Hymenoptera: Formicidae) in Taiwan
Source: Int J Environ Res Public Health. 2021 May 11;18(10):5055. doi: 10.3390/ijerph18105055 (PMC8151706; doi:10.3390/ijerph18105055)
Supplement: Supplementary file 1 [file ijerph-18-05055-s001.zip › ijerph-1192811-supplementary.pdf]

Table S1. Association of seeking medical care and symptomology

		Seeking medical care (percentage)		χ^2	*df*	*p*
Characteristics	Total record	No (%)	Yes (%)			
**Wheal-and-flare reaction**				46.90	1	0.000
No	3017	2835(94.0%)	182(6.0%)			
Yes	802	696(86.8%)	106(13.2%)			
**Anaphylactic shock**				535.06	1	0.000
No	3713	3495(94.1%)	218(5.9%)			
Yes	106	36(34.0%)	70(66.0%)			
**Cellulitis or Urticaria**				1.72	1	0.399
No	3798	3510(62.4%)	288(37.6%)			
Yes	21	21(100.0%)	0(0.00%)			
Number of Person Stung by RIFA	3819	3541(92.5%)	288(7.5%)			

Table S2. Population Density and Total Area for Counties and Cities
End of 2020

Unit : Person ; Per Square Kilometer

區域別 District	年底人口數 Year-end Population	土 地 面 積 （平方公里） Area (Sq. Km.)	人口密度 Population Density
**總　計 Grand Total**	**23,561,236**	**36,197.0669**	**651**
**新北市 New Taipei City**	**4,030,954**	**2,052.5667**	**1,964**
**臺北市 Taipei City**	**2,602,418**	**271.7997**	**9,575**
**桃園市 Taoyuan City**	**2,268,807**	**1,220.9540**	**1,858**
**臺中市 Taichung City**	**2,820,787**	**2,214.8968**	**1,274**
**臺南市 Tainan City**	**1,874,917**	**2,191.6531**	**855**
**高雄市 Kaohsiung City**	**2,765,932**	**2,951.8524**	**937**
**臺灣省 Taiwan Province**	**7,043,545**	**25,110.0037**	**281**
宜蘭縣 Yilan County	453,087	2,143.6251	211
新竹縣 Hsinchu County	570,775	1,427.5369	400
苗栗縣 Miaoli County	542,590	1,820.3149	298
彰化縣 Changhua County	1,266,670	1,074.3960	1,179
南投縣 Nantou County	490,832	4,106.4360	120
雲林縣 Yunlin County	676,873	1,290.8326	524
嘉義縣 Chiayi County	499,481	1,903.6367	262
屏東縣 Pingtung County	812,658	2,775.6003	293
臺東縣 Taitung County	215,261	3,515.2526	61
花蓮縣 Hualien County	324,372	4,628.5714	70
澎湖縣 Penghu County	105,952	126.8641	835
基隆市 Keelung City	367,577	132.7589	2,769
新竹市 Hsinchu City	451,412	104.1526	4,334
嘉義市 Chiayi City	266,005	60.0256	4,432
**福建省 Fuchien Province**	**153,876**	**180.4560**	**853**
金門縣 Kinmen County	140,597	151.6560	927
連江縣 Lienchiang County	13,279	28.8000	461
**東沙群島 Dongsha Islands**	…	**2.3800**	…
**南沙群島 Nansha Islands**	…	**0.5045**	…

Notes : 1.Population density is a measure of people per unit.

Table S3. Mean Temperature, Precipitation and Relative humidity of Taipei (30 years average, CWB, Taiwan

Component	Jan	Feb	Mar	Apr	May	Jun	Jul	Aug	Sep	Oct	Nov	Dec	Total
Temperature (°C)	16	15.9	18	21.7	24.7	27.4	29.2	28.8	27.1	24.3	21	17.6	22.6
Days of Precipitation (day)	15	15	16	15	16	15	12	14	13	12	14	13	170
Precipitation (mm)	87	166	180	183	259	319	248	305	275	139	86	78.8	2325
RH (%)	79	82	81	79	80	79	74	76	77	77	77	77	78
